# Medicinal Plant Leaf Extract and Pure Flavonoid Mediated Green Synthesis of Silver Nanoparticles and their Enhanced Antibacterial Property

**DOI:** 10.1038/s41598-017-15724-8

**Published:** 2017-11-20

**Authors:** Siddhant Jain, Mohan Singh Mehata

**Affiliations:** Laser-Spectroscopy Laboratory, Department of Applied Physics, Delhi Technological University, Bawana Road, Delhi, 110042 India

## Abstract

The rewards of using plants and plant metabolites over other biological methods for nanoparticle synthesis have fascinated researchers to investigate mechanisms of metal ions uptake and bio-reduction by plants. Here, green chemistry were employed for the synthesis of silver nanoparticles (AgNPs) using leaf extracts of *Ocimum Sanctum* (Tulsi) and its derivative quercetin (flavonoid present in Tulsi) separately as precursors to investigate the role of biomolecules present in Tulsi in the formation of AgNPs from cationic silver under different physicochemical conditions such as pH, temperature, reaction time and reactants concentration. The size, shape, morphology, and stability of resultant AgNPs were investigated by optical spectroscopy (absorption, photoluminescence (PL), PL-lifetime and Fourier transform infrared), X-ray diffraction (XRD) analysis, and transmission electron microscopy (TEM). The enhanced antibacterial activity of AgNPs against E-Coli gram-negative bacterial strains was analyzed based on the zone of inhibition and minimal inhibitory concentration (MIC) indices. The results of different characterization techniques showed that AgNPs synthesized using both leaf extract and neat quercetin separately followed the same optical, morphological, and antibacterial characteristics, demonstrating that biomolecules (quercetin) present in Tulsi are mainly responsible for the reduction of metal ions to metal nanoparticles.

## Introduction

Nanomaterials can provide solutions to many technological and environmental challenges in the field of solar energy conversion, medicine, and wastewater treatment^1^. In the process of global efforts to reduce hazardous waste, there is always need to develop a synthesis route which is economical, cost effective, non-toxic and productive. Green approach is a technique for the controllable synthesis of nanoparticles of well-defined size and shape.

Over the past few decades, there have been an increased emphasis on synthesis of metal nanoparticles and quantum dots^2,3^ because of their unique optical and electrical properties. The surface plasmon resonance (SPR) exhibited by the metal nanoparticles is one of their most important characteristics, and makes them unique with this optical properties. Metal nanoparticles proved to be very efficient and useful in the field of electronics, photonics, and medicine^4–6^. The properties of metal nanoparticles vary according to their size, shape, and morphology^7^. Nanoparticles and nanocomposites have been synthesized by chemical precipitation, solid state and ligand assisted approaches. The resultant nanostructures exhibit unique properties and are employed in various applications. There are various reports of synthesis of metal vanadate/pyrovanadate nanoparticles and nanocrystals and are tested for their influence on flame retardancy on polymeric nanocomposites^8^ and enhanced photocatalytic activity^9–12^. The applications emerge from the fact that nanoscale dimensions show different properties as compared to their bulk counterpart due to their high surface to volume ratio. This is the main reason for the development of various nanostructures, e.g., 0D, 1D, 2D and 3D arrays in a controlled manner.

Many researchers have developed a keen interest in the synthesis of silver nanoparticles due to their enhanced antimicrobial activity and their use as anticancer agents^13^. Silver in pure form has the highest electrical and thermal conductivity among all metals and has lowest contact resistance^14^. There are studies and reports that nano-silver can evidently have adverse effects on humans as well as on the environment^15^. However, the green approach offers toxic chemical free and eco-friendly synthesis of AgNPs. There are various reports of using green, i.e., natural and environment friendly reducing and capping agents for nanomaterial synthesis^16,17^. Leaves of different plants such as *Azadirachta indica* (neem)^18^, *Ocimum tenuiflorum* (black Tulsi)^19^, *Ficus benghalensis* (Banyan tree)^20^ *etc*. have been used for the synthesis of AgNPs. From over thousand years, Tulsi leaves are believed to have medicinal properties. It has miraculous healing properties mainly due to the presence of essential oils and phytonutrients. Tulsi is an excellent antibiotic, germicidal, antifungal and disinfectant, which on intake enhance the immunity of human body against various bacterial, fungal and viral infections. Not only leaves but other parts of the plants such as stem, roots, *etc*.^21^ have also been used for synthesis. Various microorganisms like bacteria, fungi, yeasts^22^ and nuclear material such as DNA^23^ have also been explored for the green synthesis of AgNPs. Besides silver nanoparticles, these micro-organisms and DNA have been employed for the synthesis of quantum dots, e.g., synthesis of CdS quantum dots (QDs) using fungi^24^.

Recently, two new biosynthetic sources, Pomegranate peel extract and cochineal dye were employed and reported to obtained AgNPs^25^. Another natural source for feasible bioreduction of metal ions to metal nanoparticles is by the use of flavonoids. A whole class of flavonoids is found in abundance in a variety of plants and derived plant products. Quercetin (molecular formula C_15_H_10_O_7_) is a polyphenolic flavonoid found in many fruits, vegetables, leaves, and grains. It can be used as an ingredient in supplements, beverages, and foods, and is an excellent antioxidant. To the best of our knowledge, there are very few reports on the synthesis of silver nanoparticles using pure flavonoid reduction, which makes the mechanism of metal ions uptake by plants and their reduction to nanostructures less explored. Therefore, we have examined the optical, morphological and antibacterial characteristics of AgNPs synthesized using both Tulsi leaf extract and neat quercetin under different physicochemical conditions. This is with a view to verify whether the obtained particles using only quercetin as precursor exhibit the same characteristics as that of particles obtained using Tulsi leaf extract. Perhaps, this investigation might provide basis for understanding the exact mechanism of how biomolecules present in plants interact with metal ions and unravel the process of their transformation into nanostructures.

## Results and Discussion

### Effect of different physicochemical conditions on the formation of AgNPs

**Figure 1:**
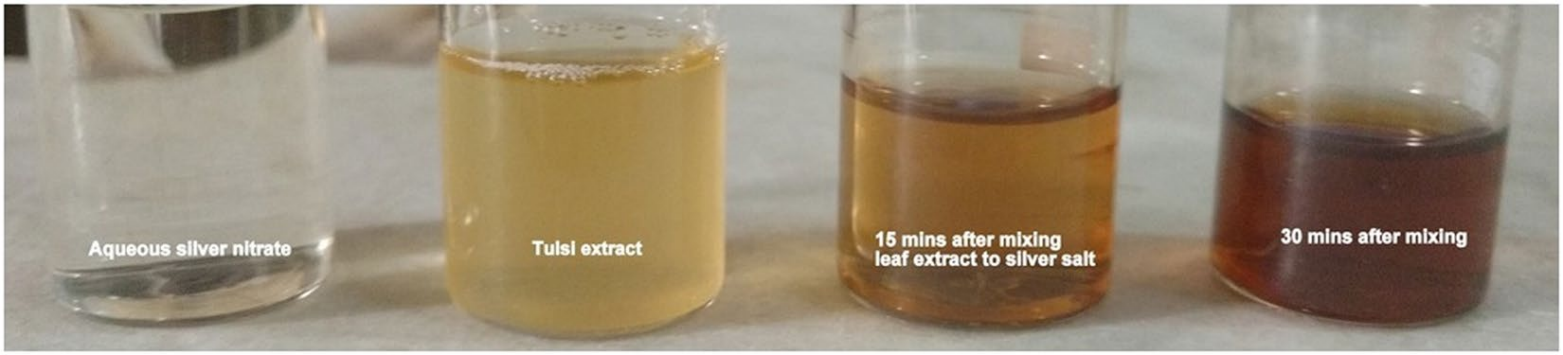
Change in the colour of the solution with time when plant extracts are added to silver salt.

**Figure 2:**
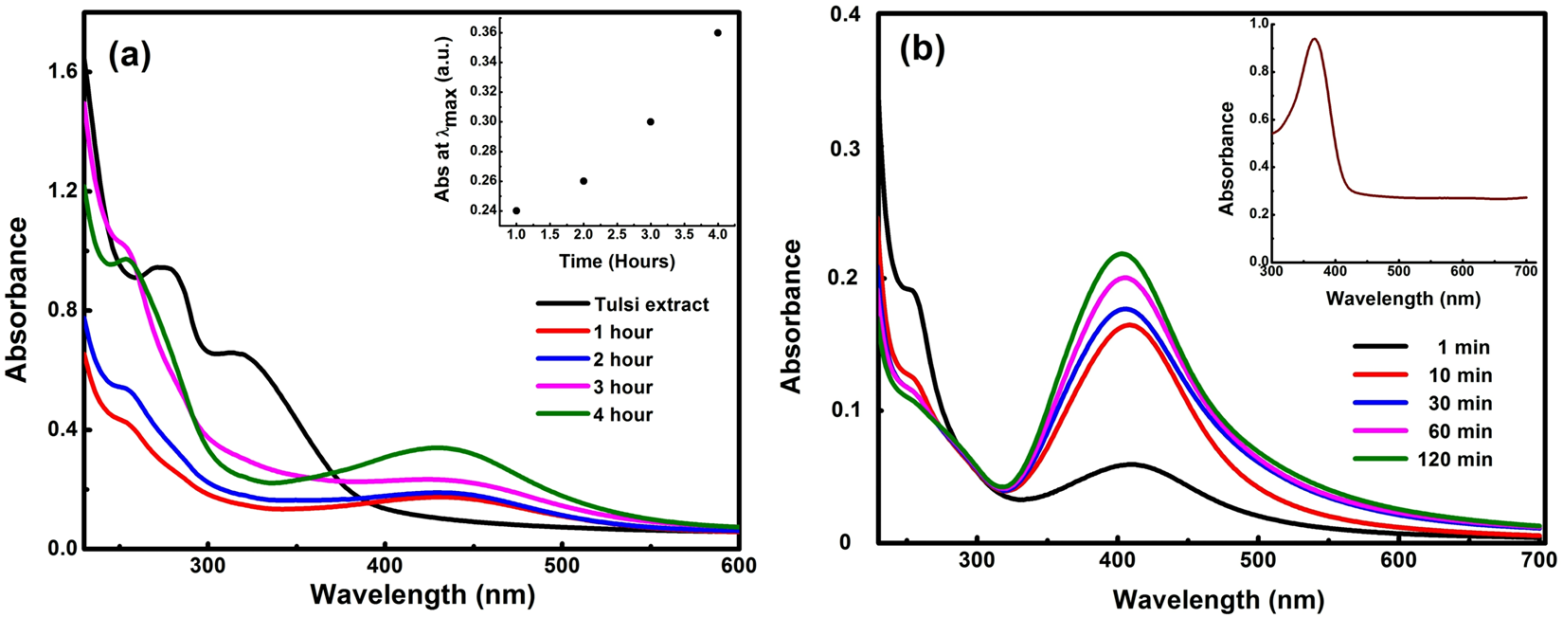
Absorption spectra of AgNPs observed at different time intervals by mixing Tulsi leaf extract **(a)** and aqueous quercetin **(b)** in 2 mM aqueous solution of silver nitrate. The inset of **(a)** shows the variation of optical intensity with time and inset of **(b)** shows the absorption spectra of neat quercetin in water.

Various environmental and physicochemical conditions such as pH, temperature, reaction time and reactants concentration significantly affect the size, shape, and morphology of AgNPs. As the plant leaf extract was mixed with the AgNO_3_ solution, a colour change from pale yellow to dark yellow and finally colloidal brown was observed within few minutes. Figure 1 shows the colour change due to the formation of AgNPs. In aqueous solution, silver nanoparticles exhibit strong surface plasmon resonance (SPR)^26^. The obtained AgNPs emits light between 400–700 nm depending on the size, shape, and morphology^27^. The bio-molecules such as flavonoids, terpenoids, phenolic compounds present in Tulsi are responsible for the reduction of silver ions to AgNPs. After few hours (hrs), there was no further change in the colour of the solution indicating that the whole silver salt present in the solution had been reduced. The formation of silver nanoparticles was examined by measuring the absorption spectra at regular time intervals. Figure 2(a) shows the absorption spectra of synthesized AgNPs in the range of 250–600 nm for the reaction of silver salt by Tulsi extract at different time intervals. Similarly, Fig. 2(b) shows the absorption spectra in the range of 250–700 nm obtained at different time intervals using quercetin as a reducing agent for the synthesis of AgNPs. An increase in absorbance was observed with the passage of time indicating an enhancement in the formation of AgNPs. It was noticed that the time required for the reduction of silver ions to silver nanoparticles was much less in the case of quercetin compared to plant extract, this fact can be explained by taking into account the structural property of quercetin. The structure of quercetin includes an extended system of conjugated double bonds and contains five hydroxyl groups which provide the high reductive ability^28^. Plant leaf extract of Tulsi contains a variable amount of phenolic compounds. The amount of quercetin in Tulsi was found to be 0.74 mg/ml of extract recorded with high-performance thin-layer chromatographic (HPTLC) method. However, the total phenolic and flavonoid content in Ocimum sanctum was 82.02 ± 8.17 mg GAE/g (where GAE is Gallic acid equivalent) and 74.6 ± 5.1 mg/g, respectively^29^.

The size of AgNPs was calculated using the following equation^30,31^.

1 $$d=\frac{h{v}_{f}}{\,\pi {\rm{\Delta }}{E}_{1/2}}$$

where *d* is the diameter of the particle, *h* is the Planck’s constant, ν_*f*_ (1.39 × 10^6^ m/sec) is the Fermi velocity of electrons in bulk silver and ΔE_1/2_ is the full width at half maximum (FWHM) of the absorption band. The above equation is valid as long as the size of the silver nanoparticles is smaller than the mean free path of the electrons in the bulk silver^32,33^. The sizes of the obtained AgNPs were calculated from the absorption spectra using eq. 1. The minimum sizes of AgNPs synthesized using tulsi extract and quercetin as calculated from the above formula came out to be about 16 and 12 nm, respectively.

**Figure 3:**
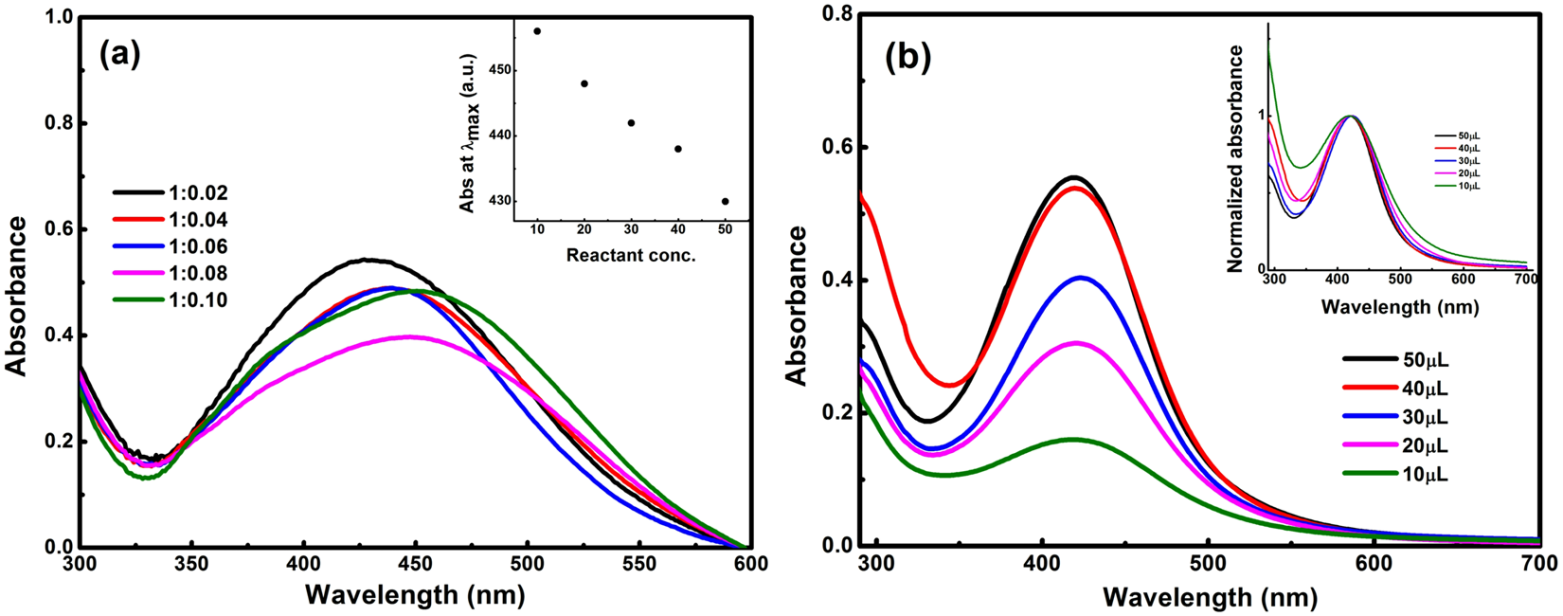
**(a)** Absorption spectra of AgNPs at various volume ratio of aqueous silver salt and Tulsi broth and the inset figure shows the shift in absorption peak with reactant concentration. **(b)** Absorption spectra of AgNPs at different amount of quercetin mixed in 5 ml aqueous solution of AgNO_3_ (concentration 2 mM). The inset figure shows the variation in spectral width with a change in concentration.

The effect of concentration of plant extract and quercetin on the formation of AgNPs was studied systematically by mixing 2 mM AgNO_3_ solution and Tulsi extract in different concentration ratios (here concentration indicates liquid volume ratio of aqueous AgNO_3_ to plant extract), i.e., 5:0.1, 5:0.2, 5:0.3, 5:0.4 and 5:0.5 (ml:ml). Similarly, a solution of silver nitrate and quercetin was prepared by mixing 2 mM aqueous AgNO_3_ with 1 mM quercetin solution in different volume ratios i.e. 5:10, 5:20, 5:30, 5:40 and 5:50 (ml:µl). On increasing the concentration of plant extract into AgNO_3_ solution, a red shift in the absorption band to the longer wavelength (from 430 to 455 nm in case of Tulsi extract & from 420 to 425 nm in case of quercetin) was observed, as shown in Fig. 3(a,b), indicating that the particle size is increasing with increasing the ratio of leaf extract and quercetin separately to the AgNO_3_ solution. In the case of quercetin, the red shift was less compared to that of Tulsi extract, which may be due to the low concentration of quercetin in AgNO_3_ solution. Since quercetin is a pure biomolecule, therefore, the minimal concentration was sufficient to reduce the same amount of silver salt as compared to Tulsi extract. On increasing the concentration of quercetin further in aqueous silver salt, it was found that the colour of the solution turned blackish grey, and the formation of AgNPs was restricted. Also, the absorption peak got narrower with an increase in concentration. According to Mie theory, the size of nanoparticles is inversely proportional to the FWHM as explained in Eq. (1), hence narrow absorption spectra, smaller FWHM indicated an increase in the size of synthesized AgNPs.

**Figure 4:**
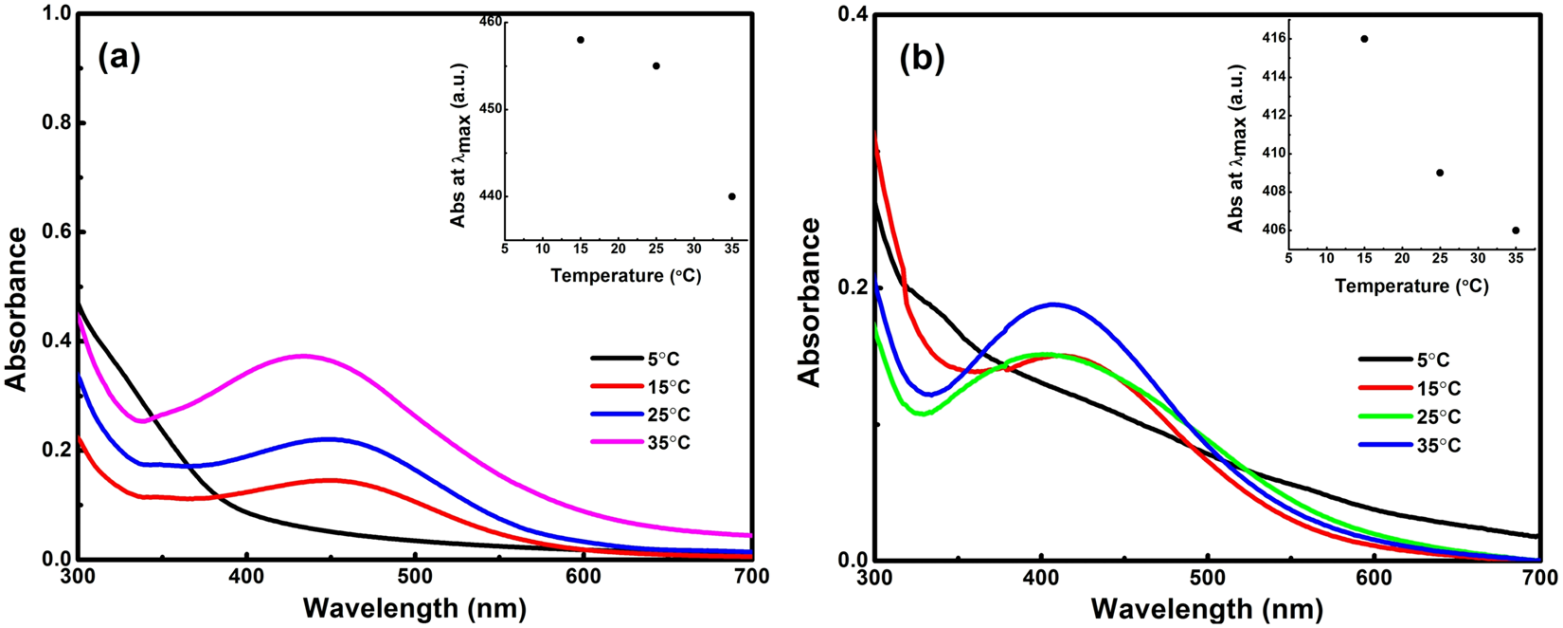
Absorption spectra of AgNPs obtained at different reaction temperatures using Tulsi extract **(a)** and quercetin **(b)** as reducing agent. Inset figure shows the relation between temperature and peak absorption wavelength **(a**,**b)**.

The reaction temperature also had significant effects on the size and morphology of the synthesized AgNPs. Figure 4(a,b) showed the variation in the absorption spectra of AgNPs synthesized at different temperature using aqueous AgNO_3_ & Tulsi extract as well as aqueous AgNO_3_ & quercetin, respectively. The reaction temperature was varied from 5–35 °C. In both cases, it was observed that with an increase in the reaction temperature the absorption peak shifted towards lower wavelength, i.e., a blue shift occurred (from 455 to 436 nm in the case of Tulsi and from 429 to 405 nm in the case of quercetin), which indicated a decrease in particle size with increase in temperature^20^. At 5 °C, there was no significant peak in the absorption spectra stating that there was no formation of AgNPs. The shift in the absorption peak was due to the localization of surface plasmon resonance of the AgNPs. This suggests that the size of the synthesized AgNPs decreases with increasing temperature, which was probably due to the faster reaction rate at a higher temperature. At high temperature, the kinetic energy of the molecules increases and silver ions gets consumed faster leaving less possibility for particle size growth. Thus, smaller particles of nearly uniform size distribution are formed at higher temperature^18^.

**Figure 5:**
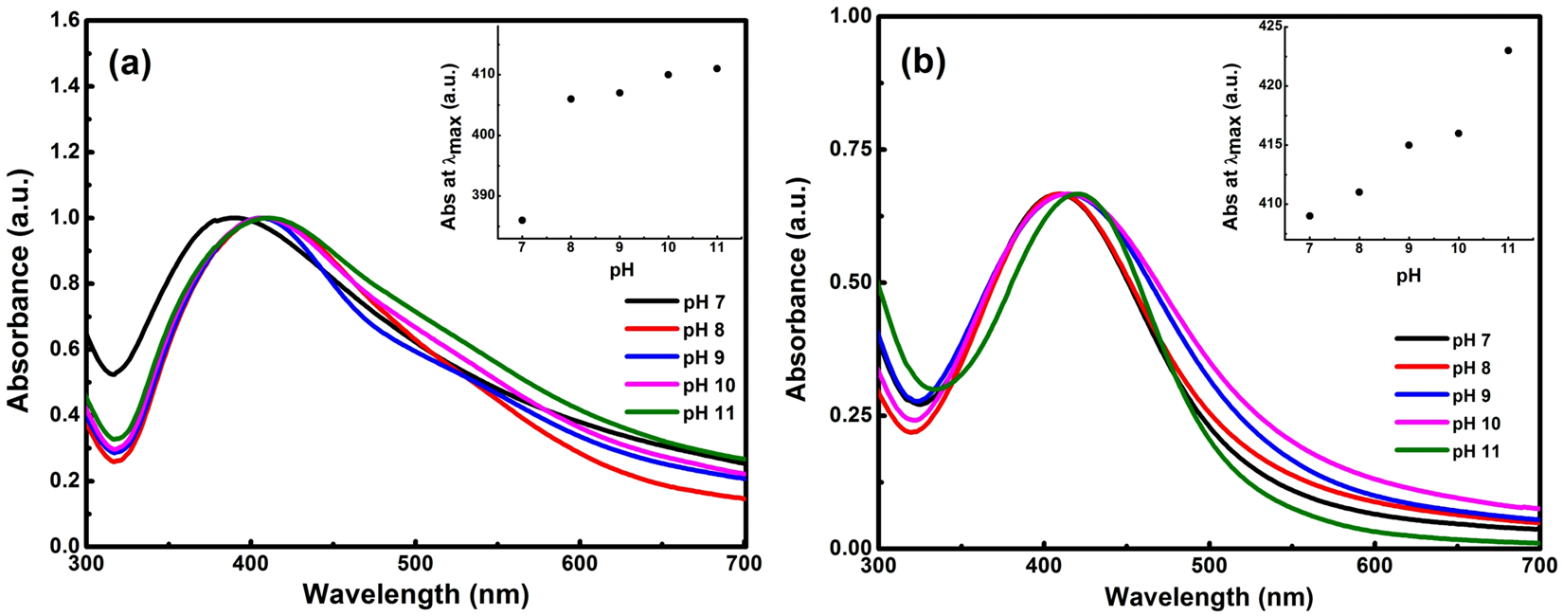
Normalized absorption spectra of AgNPs obtained at different pH values of the reaction mixture using Tulsi extract **(a)** and quercetin **(b)**. Inset figure shows the relation between pH and peak absorption wavelength maximum **(a**,**b)**.

pH is another important factor which affects the size, shape, and morphology of the synthesized AgNPs. The major influence of the reaction pH is its ability to change the electrical charges of the biomolecules (quercetin) which might change their reducing and capping ability and the subsequent growth of nanoparticles^34^. Figure 5(a,b) showed the effect of pH on the absorption spectra of AgNPs synthesized using plant extract and quercetin, respectively. In both cases, it was observed that with an increase in pH the absorption peak shifted towards higher wavelength (from 396 to 411 nm in the case of Tulsi extract & from 409 to 420 nm in the case of quercetin) indicating an increase in the size of synthesized AgNPs. The size of AgNPs synthesized using quercetin calculated from absorption spectra at higher pH (pH∼10) came out to be about 19 nm, using the formula stated in eq. (1). As the diameter of the particle increases, the energy required to excite the surface plasmon electrons decreases. As a result, the absorption maximum shifted towards longer wavelength. In addition to the spectral shift, there was an increase in the absorption intensity with increase in pH. Further, it was observed that higher pH enhances the rate of reduction as the colour of the solution turned colloidal brown more quickly as compared to a solution of lower pH. Hence alkaline pH is favorable for the synthesis of AgNPs^35^.

### Surface morphology of AgNPs

**Figure 6:**
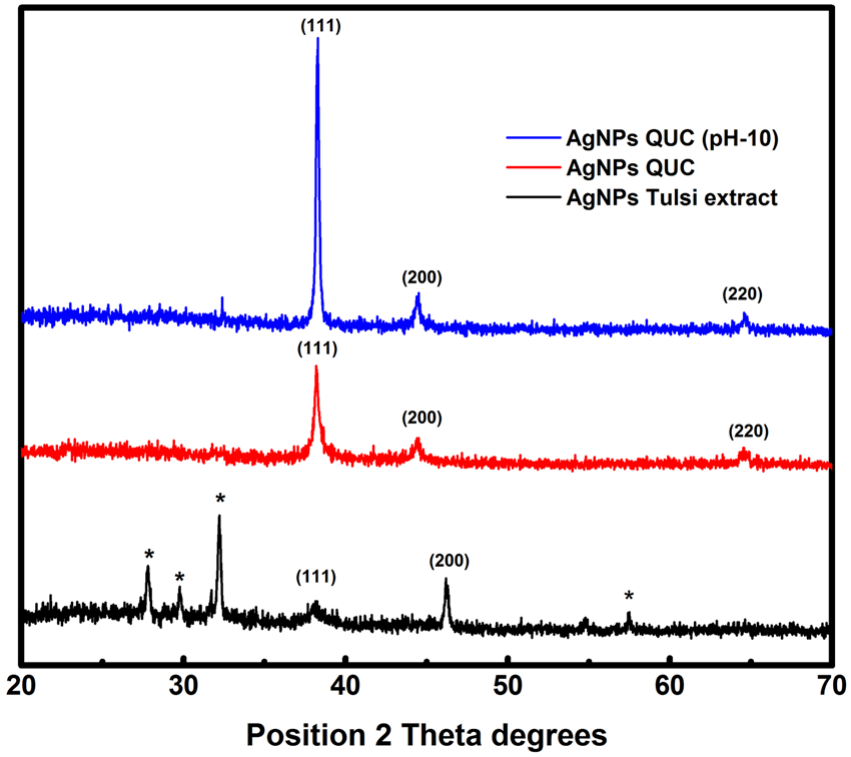
XRD patterns of AgNPs obtained using Tulsi extract and quercetin as reducing agents.

**Figure 7:**
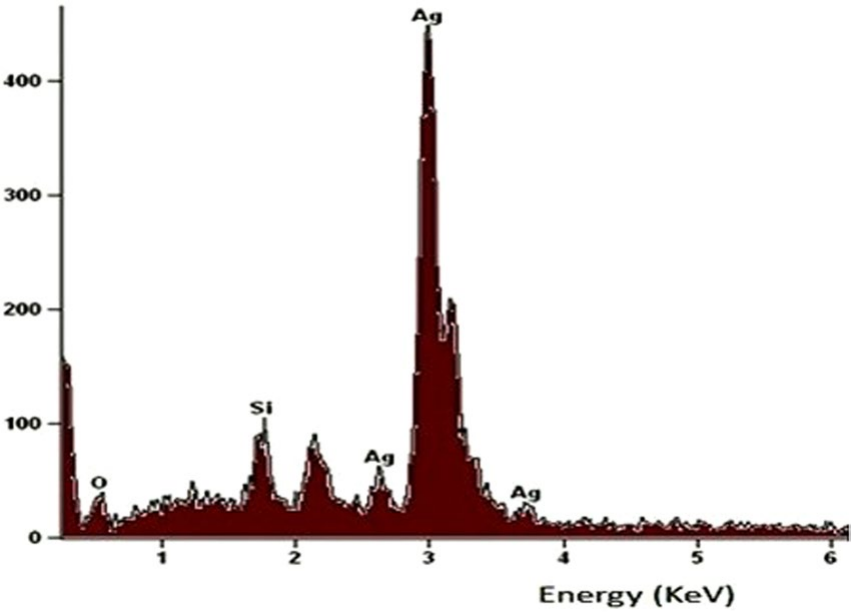
EDX spectra of synthesized AgNPs using Tulsi leaf extract as reducing agent.

To identify the crystalline nature and surface morphology of the synthesized AgNPs, X-ray diffraction (XRD) analysis was performed in the range of 30–70° at 2θ angles. Figure 6 showed the XRD pattern of AgNPs synthesized by the reaction of aqueous silver salt with Tulsi leaf extract and quercetin, respectively. The high-intensity peaks of AgNPs synthesized using quercetin were observed at around 38°, 44° and 64°corresponding to (111), (200) and (211) Bragg reflections, respectively, which were the exact peak positions as given for face center cubic (fcc) lattice structure of silver. Whereas, in the case of AgNPs obtained using Tulsi leaf extract as reducing agent the peak positions were obtained at 32°, 38°, 46° and 57°. The unassigned peaks were marked as (*), which may have occured due to the impurities present in the sample or may be related to crystalline and amorphous organic phase^36^. It can be seen from the Fig. 6 that on increasing pH of the solution the width of XRD peak decreases indicating an increase in the size of synthesized AgNPs (as the size of nanoparticles is inversely proportional to FWHM) which are in agreement with the absorption spectra of AgNPs at different pH. The average particle size obtained from XRD pattern using Debye-Scherrer equation was approximately 14 nm in the case of AgNPs synthesized using quercetin, and 17 nm in the case of AgNPs synthesized using Tulsi extract. The XRD analysis could not be performed immediately or at the same time of synthesis. Therefore, the size of AgNPs calculated from XRD spectra is bigger as compared to that calculated from absorption spectra. The energy-dispersive X-ray (EDX) analysis was performed at an accelerating voltage of 15 kV and a take-off angle of 66.6°. The quantitative results of EDX spectra of AgNPs showed a yield of 91.36 (weight %) of elemental Ag in L line. Figure 7 showed the EDX spectra of synthesized AgNPs using Tulsi extract as a precursor.

**Figure 8:**
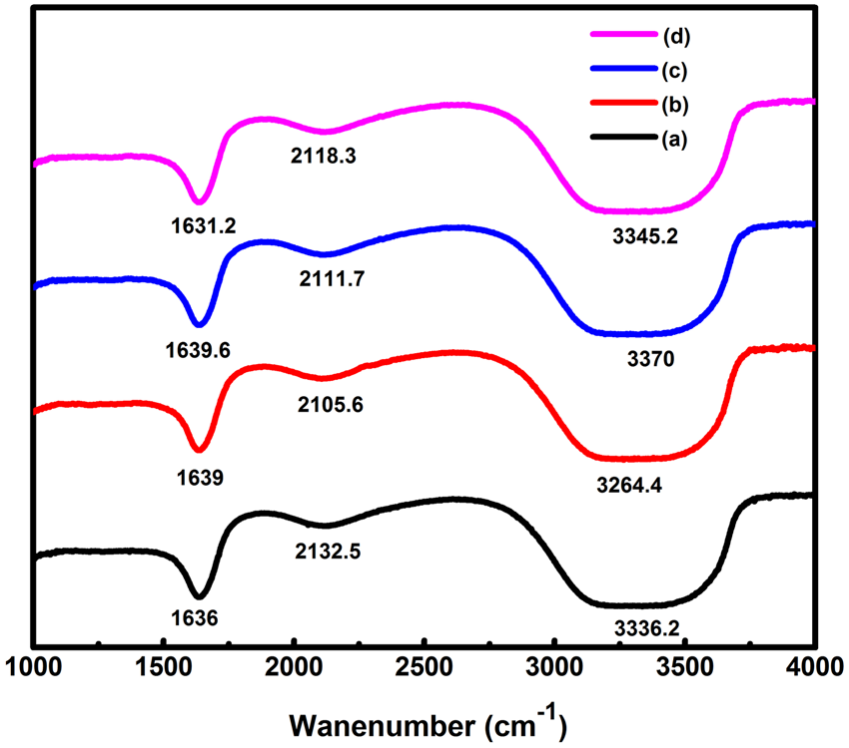
FTIR spectra of Tulsi extract **(a)**, quercetin solution in water **(b)**, AgNPs synthesized using Tulsi extract **(c)** and AgNPs synthesized using quercetin **(d)**.

The biomolecules present in plant extract responsible for the reduction of silver ions to AgNPs were identified using FTIR analysis. Figure 8 showed the FTIR spectra of pure leaf extract, quercetin solution, and AgNPs synthesized using both plant extract and quercetin as reducing agent. The FTIR spectra of fresh Tulsi extract showed peaks at around 1636, 2132 and 3336 cm^−1^ which correspond to the groups C=C (around 1635 cm^−1^), C≡C (around 2100 cm^−1^) and amine N-H/O-H vibration stretch (around 3300 cm^−1^), whereas neat quercetin showed peaks at around 1639, 2105 and 3264 cm^−1^, which correspond to the same groups like that for Tulsi extract^37^. This indicates that quercetin is a major biomolecule present in Tulsi and was responsible for the reduction of silver ions to AgNPs. The silver nanoparticles synthesized using quercetin showed peaks at around 1640, 2112 and 3370 cm^−1^ corresponding to the functional groups of amide C=O (around 1640 cm^−1^), C≡C stretch (around 2100 cm^−1^) and amine N-H/ O-H vibration stretch (around 3370 cm^−1^)^38^. In the case of AgNPs synthesized using Tulsi extract, the peaks were at around 1631, 2118 and 3345 cm^−1^, which represent the same functional groups like that for AgNPs synthesized using quercetin^39^.

**Figure 9:**
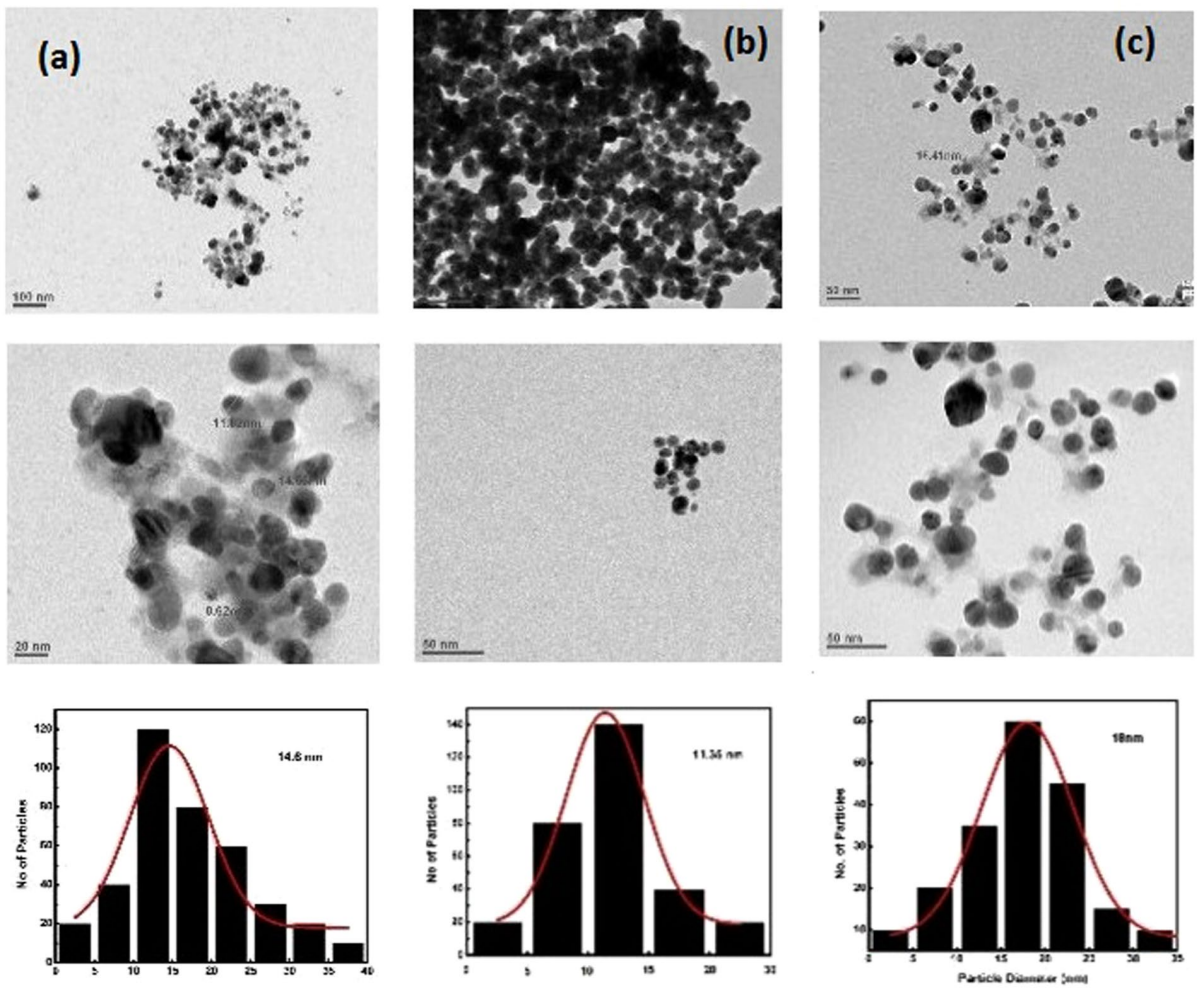
TEM micrographs and particle size distribution histogram of AgNPs synthesized using Tulsi extract **(a)**, quercetin **(b)** and quercetin at pH 10 of the reaction mixture **(c)**.

Figure 9(a–c) showed the TEM images of AgNPs synthesized using both Tulsi leaf extract and quercetin. The prepared particles were nearly spherical in shape and uniform in size distribution. The mean particle size obtained using Tulsi extract and quercetin as reducing agents were 14.6 and 11.35 nm, respectively. TEM analysis confirmed that on increasing the pH of the reaction mixture, the size of AgNPs increases. The mean particle size obtained at pH 10 was 18 nm, which is in good correlation with absorption and XRD spectra. Some particles got agglomerated in overnight as can be seen in the micrographs; therefore, the agglomerated particles were neglected in calculating the mean particle size of AgNPs.

### Stability of AgNPs

**Figure 10:**
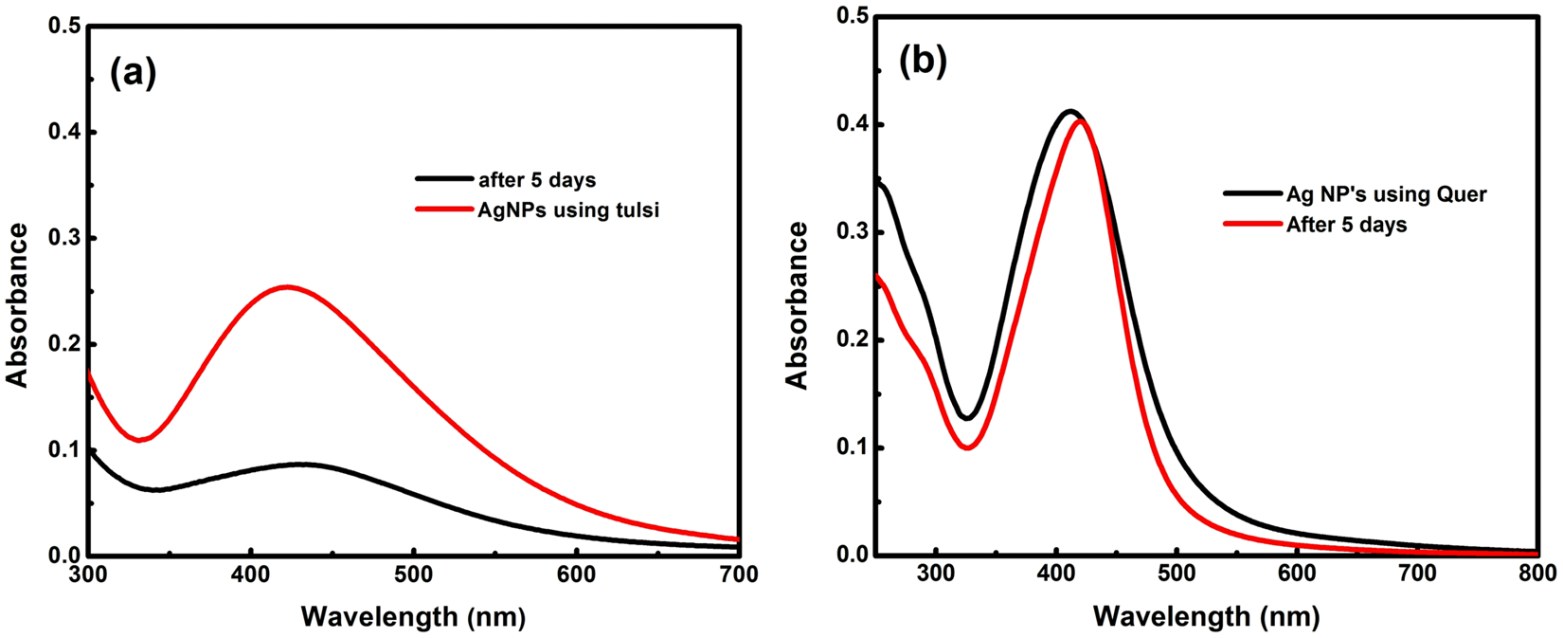
Absorption spectra of the same sample of AgNPs synthesized (at neutral pH and room temperature) using Tulsi extract **(a)** and quercetin **(b)** taken after five days.

Stability is another important parameter ensuring the area of application in which nanoparticles can be incorporated. The stability of silver nanoparticles varies as per the synthesis technique, storage conditions and also with stabilizing and capping agent used in the synthesis process. Figure 10(a,b) illustrated the change in absorption spectra of AgNPs taken after five days, indicating the shift in peak towards higher wavelength and a decrease in the absorption intensity. However, the change in absorption spectra of AgNPs synthesized using quercetin showed little variation compared to the absorption spectra of AgNPs synthesized using Tulsi leaf extract, which indicates that quercetin alone acts as a better stabilizer than Tulsi extract. However, Tulsi extract contains quercetin as the main biomolecule, but other biomolecules present in Tulsi might have low capping potential, which may hinder the overall capping ability of Tulsi extract.

Figure S1 (see supplementary information) showed the PL spectra of AgNPs containing solution obtained using Tulsi extract at different time interval with 320 nm excitation wavelength. It was seen that the PL intensity increased with time and a sharp peak was obtained at around 440 nm. The small peak observed at around 360 nm was due to the water Raman (Inset Supplementary Fig. S1). The PL spectra of AgNPs solution obtained using quercetin as a reducing agent with an excitation wavelength of 340 nm is shown in Fig. S2. The Inset Figure showed the PL spectra of quercetin solution in water and ethanol, respectively. The PL spectra of quercetin in ethanol showed no small peak whereas the PL spectra of quercetin in water showed a small additional peak at around 370 nm, which confirms that the small peak corresponds to water Raman. Since bulk silver is a metal, it has no or very narrow band gap due to which there should be no photoluminescence (PL) from silver. However, when it is top downed at the nanoscale, a band gap must have been created which may produce PL. In the case of AgNPs both interband and intraband transitions occur between the electronic states, the PL of AgNPs is due to excitation of electrons from occupied *d* band into the states above the Fermi level^40^. When surface plasmon electrons absorb light at a resonant frequency a part of this energy is transferred into heat and part of this energy is radiated as PL, and recombination occurs between electrons of *sp* band with a hole in *d* band^41^.

Further, to understand the role of biomolecules, quercetin, time-resolved PL decays were measured with 340 nm excitation for neat quercetin and Tulsi extract, and also for AgNPs samples obtained using Tulsi and quercetin (see Supplementary Figs S3 and S4). All the four decay curves are non-exponential, as could not fit well with single-exponential function. PL decay of pure quercetin fitted well with tri-exponential function $({\rm{I}}({\rm{t}})={\alpha }_{1}\exp \,(-t/{\tau }_{1})+{\alpha }_{2}\exp \,(-t/{\tau }_{2})+{\alpha }_{3}\exp \,(-t/{\tau }_{3}))$ with an average lifetime $({\tau }_{av}={{\rm{\Sigma }}}_{i}{\alpha }_{i}{\tau }_{i}/{{\rm{\Sigma }}}_{i}{\alpha }_{i})$ of 428 ps (Fig. S3). Where ${\tau }_{1},{\tau }_{2}\,$and ${\tau }_{3}$ represent the lifetimes and *α*_1_, *α*_2_ and *α*_3_ are the corresponding pre-exponential factors, respectively (Table 1). The average lifetime of Tulsi extract follow the similar trend of a triexponential fit with longer average lifetime. The tri-exponential decay of quercetin may be due to the rotation of a single bond between benzopyran rings to phenyl rings^42^. This also confirms that the major contribution in Tulsi extract is quercetin with a lifetime of 90 ps (Table 1), which is inconsistence of the reported value^42^. The lifetime of AgNPs solution is comparable in both cases. However, in the case of Tulsi extract, the average lifetime is a bit longer, which may be due to the complex formation of AgNPs with non-reducing biomolecules present in Tulsi extract.

**Table 1:** PL lifetimes and pre-exponential factors of fresh Tulsi extract, quercetin and AgNPs contained solution.

Sample	τ_1_ (ns)	τ_2_ (ns)	τ_3_ (ns)	α_1_	α_2_	α_3_	χ^2^	τ_av_ (ps)
Tulsi extract	0.09	1.95	7.61	0.94	0.03	0.03	1.19	371
Neat quercetin	0.09	2.05	12.09	0.93	0.05	0.02	1.27	428
AgNPs(Tulsi *extract*)	0.07	1.65	8.18	0.95	0.03	0.02	1.23	279
AgNPs (Quercetin)	0.73	2.99	9.18	0.80	0.17	0.03	1.16	136

### Antibacterial activity of AgNPs

**Figure 11:**
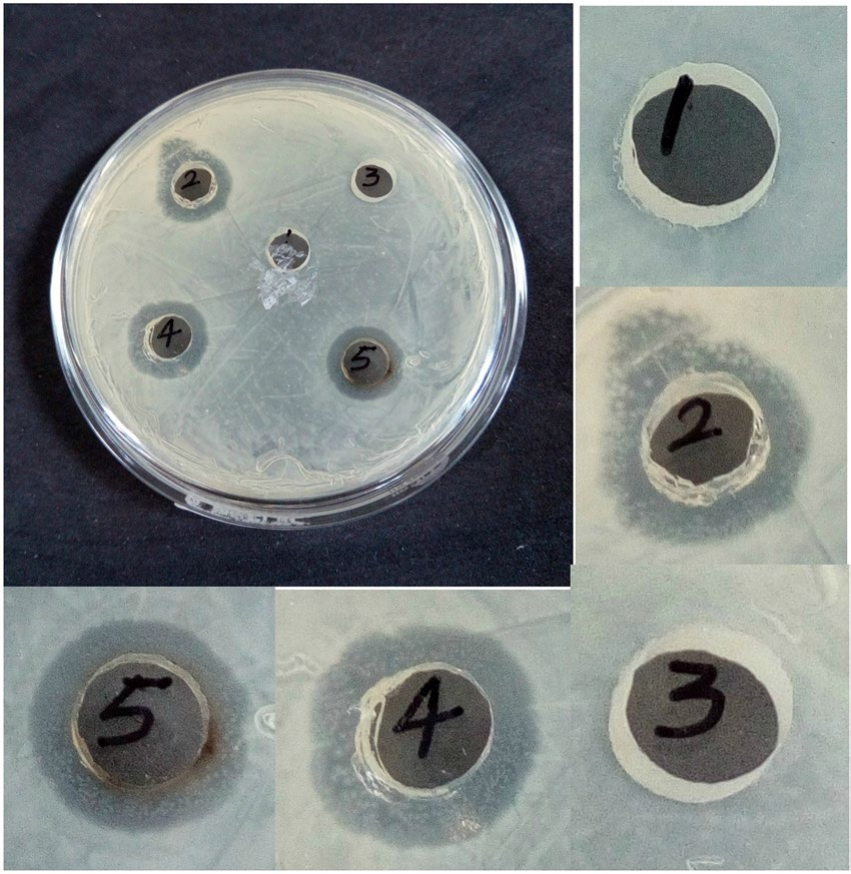
Zone of inhibition observed when gram-negative bacterial strains are treated with Tutsi extract **(1)**, AgNO_3_ **(2)**, neat quercetin **(3)**, AgNPs (Tulsi) **(4)** and AgNPs (quercetin) **(5)**.

**Figure 12:**
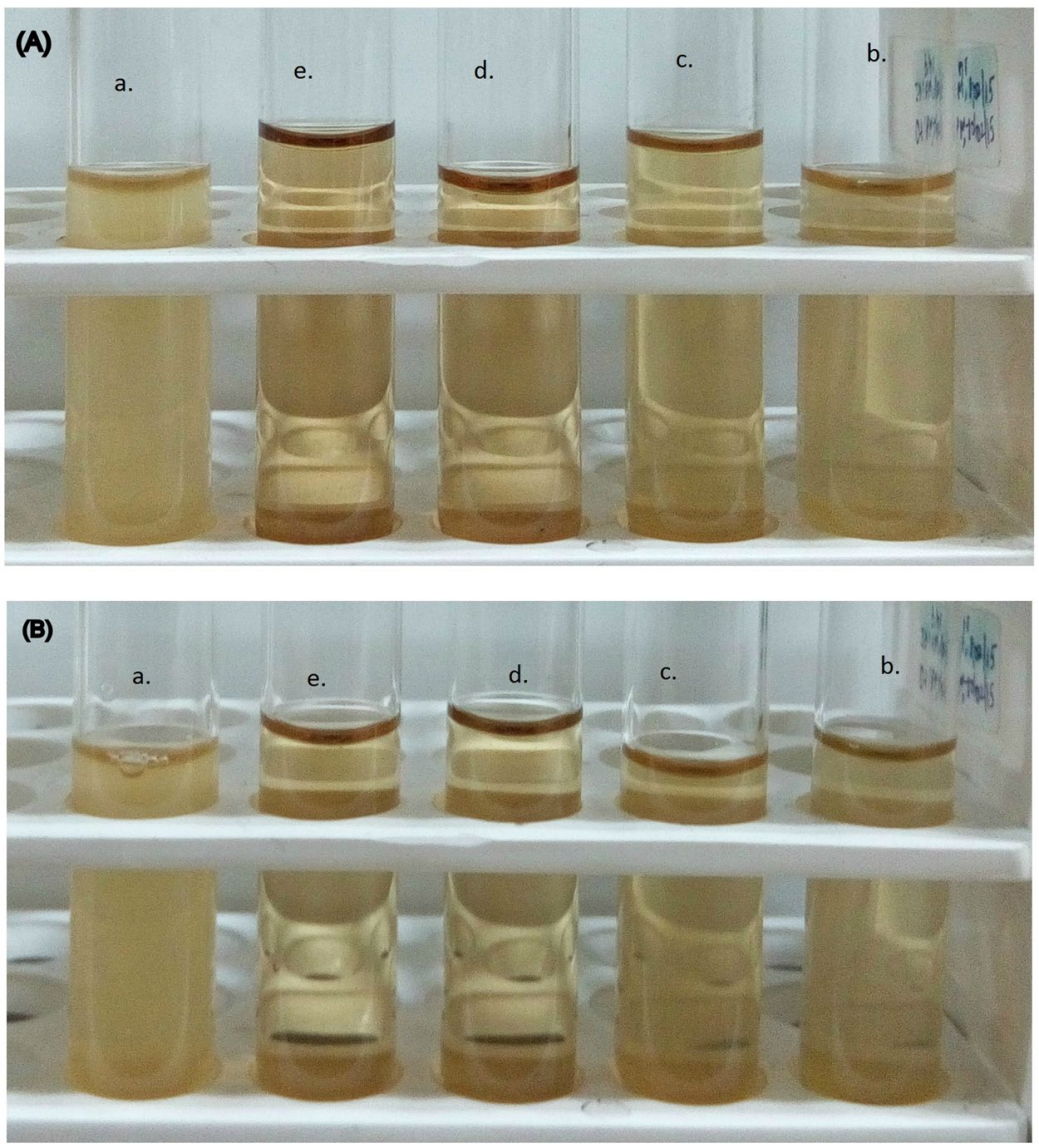
MIC analysis when gram-negative bacterial culture broth is treated with different concentration of AgNPs synthesized using quercetin **(A)** and Tulsi extract **(B);** pure bacterial culture (untreated) (a), 50 (b), 100 (c), 150 (d) and 200 μg/ml (e).

It is well known that AgNPs have a higher surface to volume ratio as compared to their bulk counterpart. Therefore, some interactions with the bacterial surfaces are facilitated, and antibacterial property of AgNPs is enhanced. Reports show that the interaction of AgNPs with the sulphur and phosphorus containing constituents of the bacterial cell initiates cell killing by attacking the respiratory chain and cell division^43^. Also, it was seen from disk diffusion method (Fig. 11) that AgNPs synthesized using both Tulsi extract and quercetin had increased antibacterial activity compared to pure Tulsi extract, aqueous quercetin and AgNO_3_ as the zone of inhibition (diameter in mm) observed in case of bacterial strain treated with AgNPs was much greater than that of bacterial strain treated with other samples. In fact, cationic silver (Ag^+^) present in aqueous AgNO_3_ also does have antibacterial effects. However, nanoparticles have a higher antibacterial activity than the free silver ions^44^. The zone of inhibition obtained when the gram-negative bacterial strain was treated with different samples (Table 2). For MIC calculation, as can be seen from Fig. 12(A,B), the gram negative bacterial samples (aqueous cultures) were treated with different concentrations (0, 50, 100, 150, and 200 µg/ml) of AgNPs obtained using both Tulsi extract and quercetin as reducing agents, respectively for about 24 hrs. It can be seen from the figures that the untreated bacterial cultures were turbid, and the cloudiness seemed to disappear with an increase in the concentration of AgNPs, and a clear solution was obtained at 150 µg/ml concentration of AgNPs synthesized using both Tulsi extract and quercetin, respectively. From both the methods employed for analyzing the antibacterial activity of AgNPs, it can be inferred that AgNPs obtained using Tulsi extract and quercetin has almost same antibacterial potential.

**Table 2:** Zone of inhibition of different samples treated against gram-negative bacterial strains.

Samples	Zone of inhibition in (mm)
Tulsi extract	—
Aqueous AgNO_3_	10
Neat quercetin	—
AgNPs (Tulsi extract)	14
AgNPs (quercetin)	14

### Probable mechanism for synthesis of AgNPs

**Figure 13:**
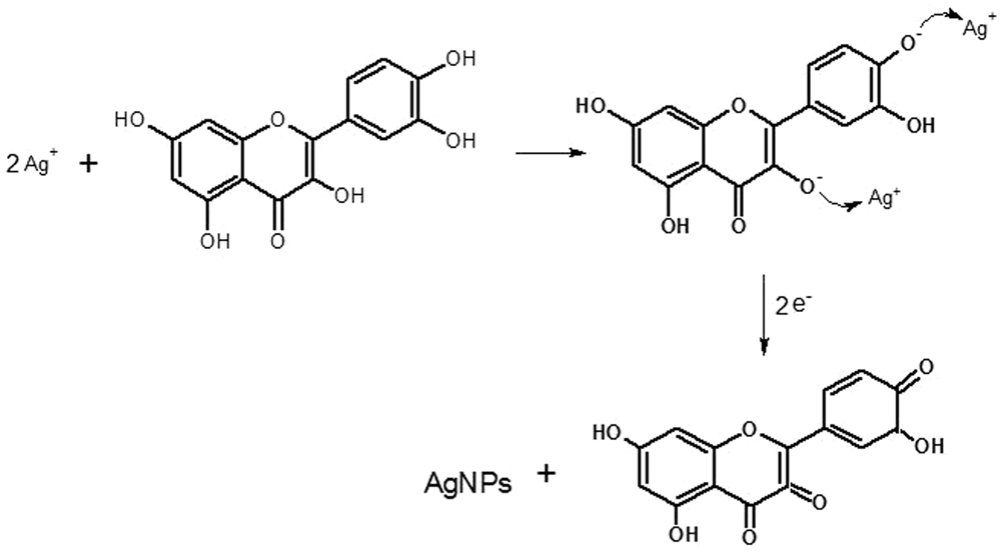
Mechanism of reduction of silver ions to AgNPs by quercetin molecule.

**Figure 14:**
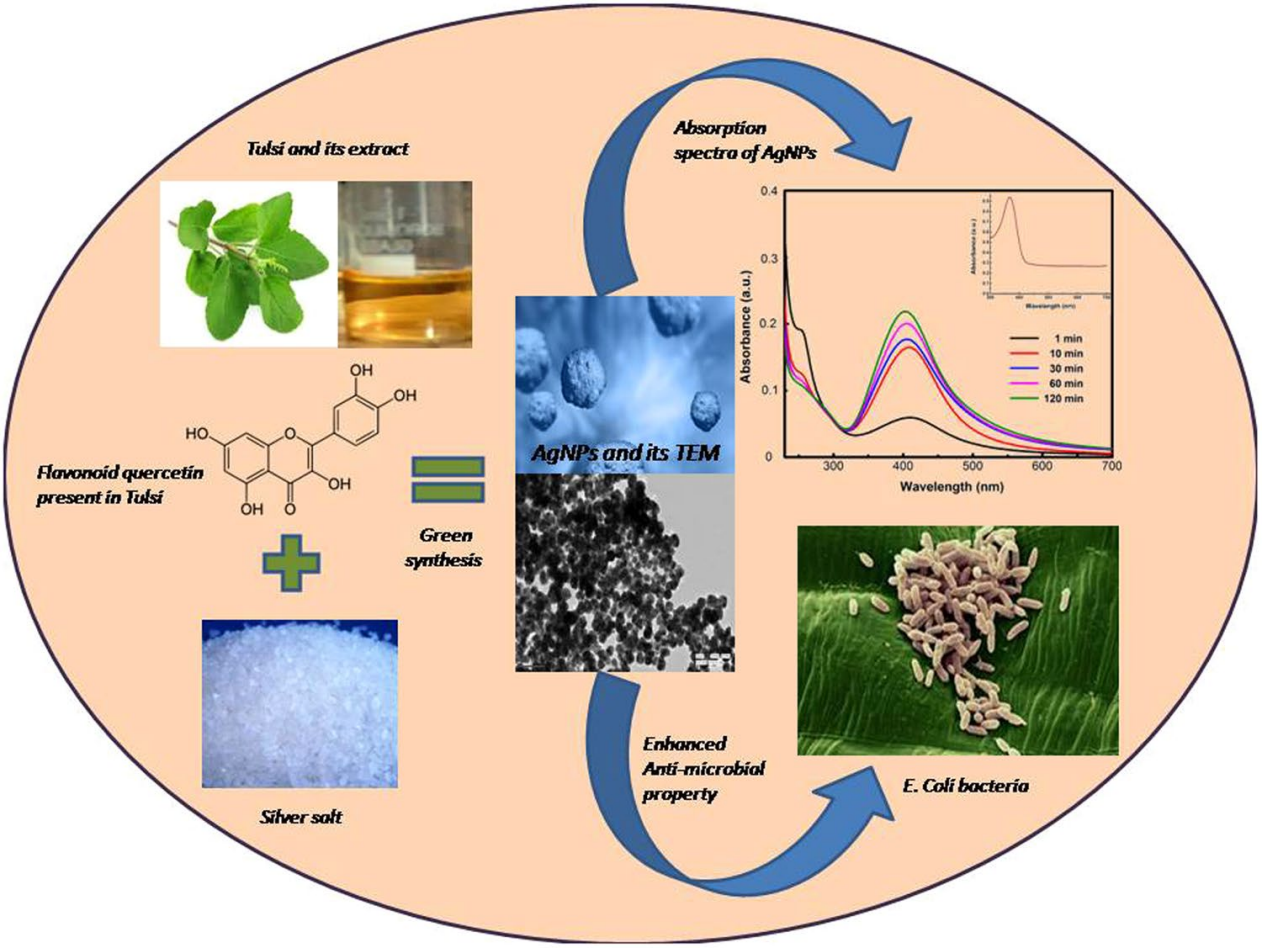
Schematic representation of the synthesis of AgNPs from Tulsi extract and quercetin showing strong absorption and enhanced antibacterial property.

There is no proper literature explaining the mechanism for the flavonoid reduction and stabilization of AgNPs. The mechanism of nanoparticle formation consists of mainly three stages: reduction of ions, clustering and further nanoparticle growth. The features of each stage depend upon the nature of reducing agent, its concentration, pH, AgNO_3_: reducing agent concentration^45^. According to some researchers, the -OH groups present in flavonoids such as quercetin may be responsible for the reduction of silver ions to AgNPs^46^. It is possible that the tautomeric transformation of flavonoids from enol form to keto form may release reactive hydrogen atom that reduces silver ions to silver nanoparticles. Zhang *et al*.^47^ reported that quercetin has high reduction potential, therefore, acts as a reducing agent. Figure 13 showed the proposed mechanism of synthesis of AgNPs by a flavonoid reduction of silver ions to AgNPs. DFT analysis indicated that the bond dissociation energies of O–H bond of –OH groups of catechol moiety of flavonoids are less than that of other -OH groups present in flavonoids^48^. The results indicated that -OH groups of catechol moiety of flavonoids might have taken part in metal ion reduction. The redox reaction shown in Fig. 13 illustrates the production of two protons per catechol^49^, i.e., one molecule of quercetin reduces two silver ions. As AgNO_3_ in distill water dissociates into silver ions (Ag^+^) and nitrate ions (NO_3_^−^). Tulsi contains a high amount of flavonoid (quercetin) having hydroxyl and ketonic groups. Quercetin reacts with Ag^+^ as an acid through the most reactive hydroxyl groups attached to the aromatic ring carbon atoms which can reduce the silver ions to silver nanoparticles and provide stability against agglomeration^45,50,51^. The enzymes present in leaf extract combines with silver ions to form an enzyme substrate complex with a charge transfer between quercetin and Ag^+^ resulting into formation of protein capped silver nanoparticles. Figure 14 shows the Schematic representation of the synthesis of AgNPs from Tulsi extract and quercetin showing strong absorption and enhanced antibacterial property.

## Conclusions

We have successfully compared the effects of environmental conditions such as temperature, pH, reaction time and reactants concentration on the green synthesis of AgNPs using Tulsi leaf extract and quercetin separately as reducing agents. The results obtained using different characterization techniques showed prominent similarities indicating that bio-molecules such as quercetin present in plant extracts of Tulsi, Neem, *etc*. are mainly responsible for the reduction of silver ions to AgNPs. The synthesized AgNPs exhibited strong absorption maximum between 400–450 nm depending on the size, shape, and morphology of the resultant particles. The average particle size of synthesized AgNPs obtained using the green approach varied between 10–20 nm. Environmental conditions can significantly alter the size and shape of synthesized AgNPs, hence desirable size and shape of AgNPs for various applications can be integrated easily using the green method. The synthesized AgNPs showed enhanced antibacterial property against gram-negative bacteria (E-Coli), which suggests possible bio-medical applications.

## Materials and Methods

### Synthesis of AgNPs

Silver nitrate (AgNO_3_) salt and quercetin powder (99% purity) were obtained from Sigma-Aldrich Chemical Co. and were used without further purification. All the glass wares were properly cleaned before used. Plant extract was prepared from leaves of Ocimum Sanctum (Tulsi) that were first rinsed with tap water and then distilled water to remove all the dust and unwanted visible particles. Then the leaves were dried at room temperature to remove the water from the surface of the leaves. About 2 g of finely incised dried Tulsi leaves were boiled in 40 ml distilled water at 60 °C for about 10 min. The supernatant was filtered using Whatman filter paper No.1 to remove the particulate matter. A pale yellow clear solution is obtained and stored at 4–8 °C.

The flavonoid stock solution was prepared by taking about 0.003 gm of quercetin (yellow powder) and dissolving it in 10 ml mixture of distilled water and ethyl alcohol (v/v ratio) to obtain 1 mM solution (as quercetin partially dissolves in normal distilled water). For analysis of variation in pH, it was dissolved in a different amount of 1 mM solution mixture of sodium hydroxide (NaOH) and distilled water (for analysis at pH 7–11). At normal pH, the solution was pale yellow, but at higher pH, the colour of the stock solution turned orange. The stock solution was stored at room temperature in a colored glass bottle (or wrap aluminum foil around transparent glass bottle) to prevent the photo-degradation of quercetin molecule^52^.

2 mM solution of silver nitrate was prepared by dissolving 0.0085 gm of AgNO_3_ in 25 ml of distilled water. 1 ml extract of Tulsi leaf was mixed with 5 ml of 2 mM AgNO_3_ solution. Similarly, about 50 µM quercetin (a flavonoid) solution was mixed with 5 ml of 2 mM AgNO_3_ solution. A colour change from pale yellow to colloidal brown indicated the formation of silver nanoparticles. The effects of various parameters such as pH, concentration, reaction time and temperature on the synthesis of silver nanoparticles were examined.

Effect of time was studied at regular intervals of 1, 2, 3 and 4 h in the case of leaf extract and 1, 10, 30, 60 and 120 min while using quercetin as a precursor. The disparity in the time interval for the above is discussed in the result section. Effect of pH was studied by varying the pH of leaf broth and quercetin solution. The pH of the AgNO_3_ solution was not varied. An amount of 0.1 N NaOH and 0.1 N HCl were used to adjust the pH of the leaf extract and quercetin solution, and the pH of the sample varied from pH 7–11 with an accuracy of ± 0.2. Effect of temperature was studied by varying the temperature between 5–35 °C for both Tulsi extract and quercetin with an accuracy of ± 2 °C.

For antibacterial activity, the gram-negative (E-Coli) bacterial cultures were prepared by the standard process. The petri-dish and agar were autoclaved before use. Tulsi leaf extract, aqueous quercetin and silver nanoparticle samples were exposed to UV radiations for 1 h to remove any unwanted bacterial impurity. Zone of inhibition analysis was carried out using disk diffusion method in which 0.1 ml of pure bacterial cultures were uniformly spread on nutrient agar plates using an L-rod. The media was then punched with 6 mm diameter holes and 20 µL aqueous samples of Tulsi leaf extract, neat quercetin and AgNPs were transferred into the wells. The results were obtained by incubating the sample for 48 hours at 35 °C.

For minimal inhibitory concentration (MIC) analysis, the bacterial culture broths were treated with different concentrations of AgNPs obtained using both Tulsi extract and quercetin separately as reducing agents. MIC is the lowest concentration of an antibacterial agent which completely inhibits the visible growth of a microorganism after incubating overnight. Pure bacterial culture broth is turbid when seen with naked eyes. The bacterial cultures were treated with the different concentrations of AgNPs to observe the minimum concentration of AgNPs at which the turbidity was totally eliminated, and a clear solution was obtained.

### Characterization of synthesized AgNPs

#### Optical Spectroscopy

The absorption spectra of aqueous precursors and synthesized AgNPs were recorded with UV/VIS/NIR spectrometer (Perkin Elmer Lambda 750) and photoluminescence spectra were recorded with Fluorolog-3 Spectrofluorometer (Horiba Jobin Yvon) equipped with double-grating excitation and emission monochromators (1200 grooves/mm) and R928P photomultiplier tube (PMT). The excitation source was a 450-Watt CW Xenon lamp. Photoluminescence decays were recorded using time-correlated photon counting (TCPC) system (DeltaFlex-01-DD, Horiba Jobin Yvon IBH Ltd) coupled with DeltaDiode (340 nm) and PMT (PPD 850) and the data were analyzed using reconvolution and least square fitting method using the commercial software provided by Horiba. The time resolution of the equipment is typically <50 ps with the DeltaDiode laser using reconvolution method.

#### FTIR analysis

The chemical compositions of plant extract, quercetin and the synthesized silver nanoparticles were studied using FTIR spectrometer (Thermo Scientific Nicolet 380). The solutions were characterized in the range 4000–500 cm^−1^ using KBr pellet.

#### XRD and EDX Analysis

The samples for XRD and EDX analysis were prepared by carefully depositing a thin film of silver nanoparticles on a glass slide by injecting and heating the AgNPs solution drop by drop at 60 °C allowing the solvent to evaporate. The crystalline nature and surface morphology of synthesized nanoparticles were studied. The EDX images were recorded by Hitichi-640 integrated with scanning electron microscope (SEM) at accelerating voltage of 15 KV and XRD pattern were recorded by BRUKER- D8 Advanced (Cu Kα radiation at a voltage of 30 kV and current of 20 mA). Different phases present in the synthesized samples were determined by the JCPDS software using search and match facility. The average particle size of the prepared samples was determined by using Scherrer’s equation as follows; D = 0.94 λ/β cosθ, where D is the crystal size, λ is the wavelength of X-ray, θ is the Braggs angle in radians and β is the FWHM of the peak in radians.

#### TEM imaging

The samples for imaging were prepared by carefully placing a single drop of synthesized AgNPs (aqueous) on a copper coated grid, and the samples were imaged on TECNAI (TEM) Electron Microscope at SAIF, AIIMS, New Delhi.

## Electronic supplementary material

Supporting Information
