# Supplementary material for: Medicinal Plant Leaf Extract and Pure Flavonoid Mediated Green Synthesis of Silver Nanoparticles and their Enhanced Antibacterial Property
Source: Sci Rep. 2017 Nov 20;7:15867. doi: 10.1038/s41598-017-15724-8 (PMC5696514; doi:10.1038/s41598-017-15724-8)
Supplement: Supplementary file 1 — Supporting Information [file 41598_2017_15724_MOESM1_ESM.pdf]

Supplementary Information

Medicinal Plant Leaf Extract and Pure Flavonoid Mediated Green Synthesis of Silver Nanoparticles and their Enhanced Antibacterial Property

Siddhant Jain and Mohan Singh Mehata*

Laser-Spectroscopy Laboratory, Department of Applied Physics, Delhi Technological University, Bawana Road, Delhi, 110042. INDIA.

*Corresponding author E-mail: msmehata@gmail.com; msmehata@yahoo.com

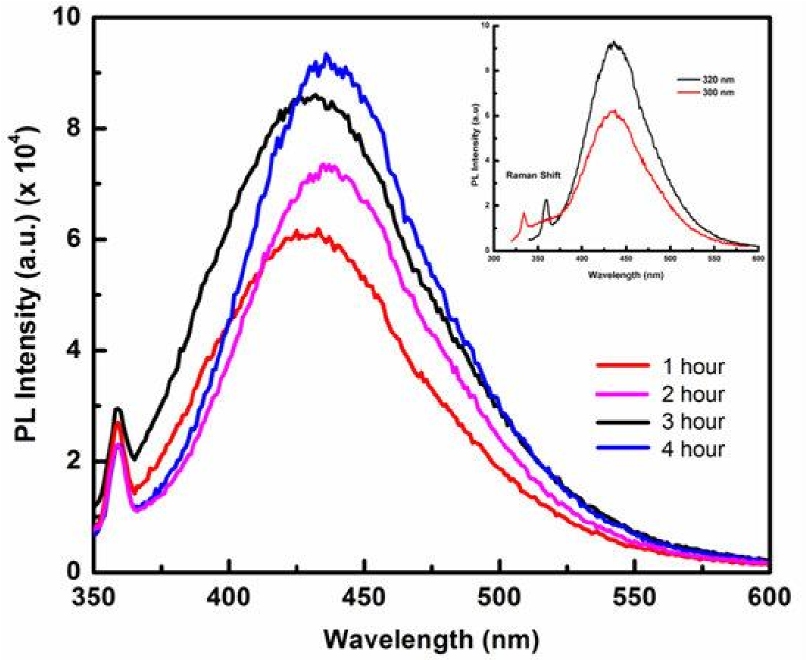

Figure S1: PL spectra of AgNPs at different time interval obtained using tulsi extract and silver salt. The inset figure shows the Raman shift of water peak (the smaller peak).

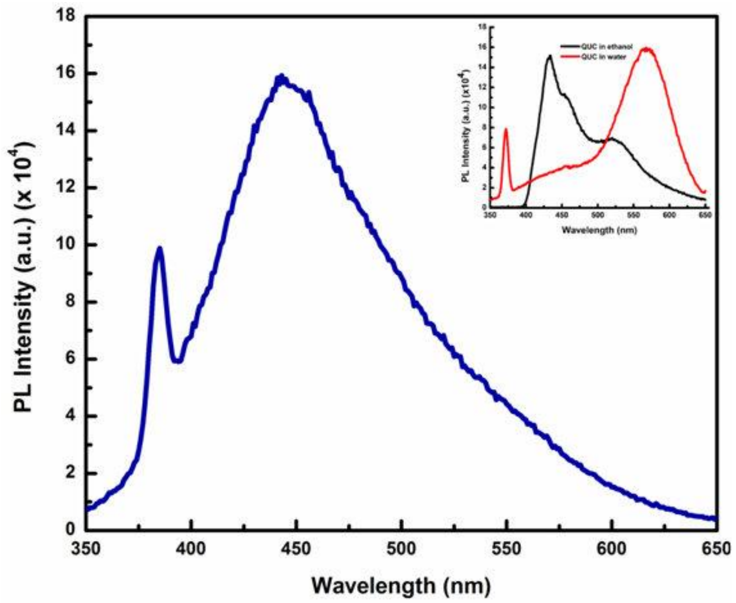

Figure S2: PL spectra of AgNPs in water, obtained using quercetin solution and a silver salt. The inset shows the PL spectra of neat quercetin in water and ethanol, respectively.

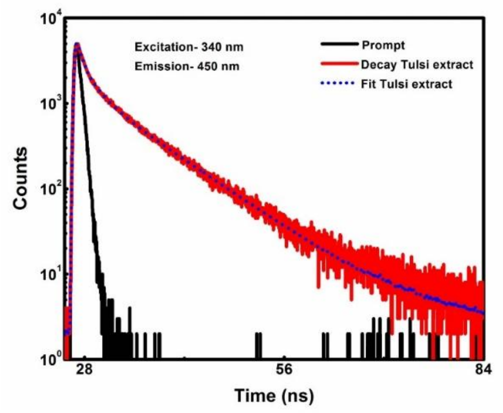

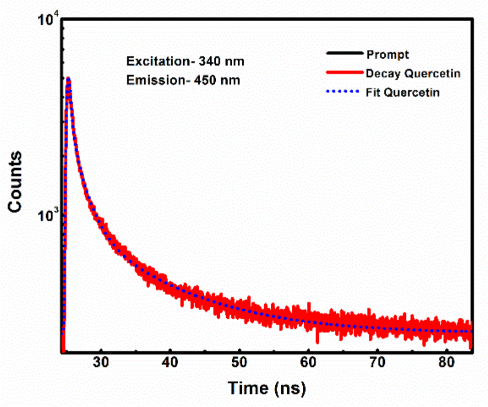

Figure S3: PL decay profiles of fresh Tulsi extract (left) and neat quercetin in water (right).

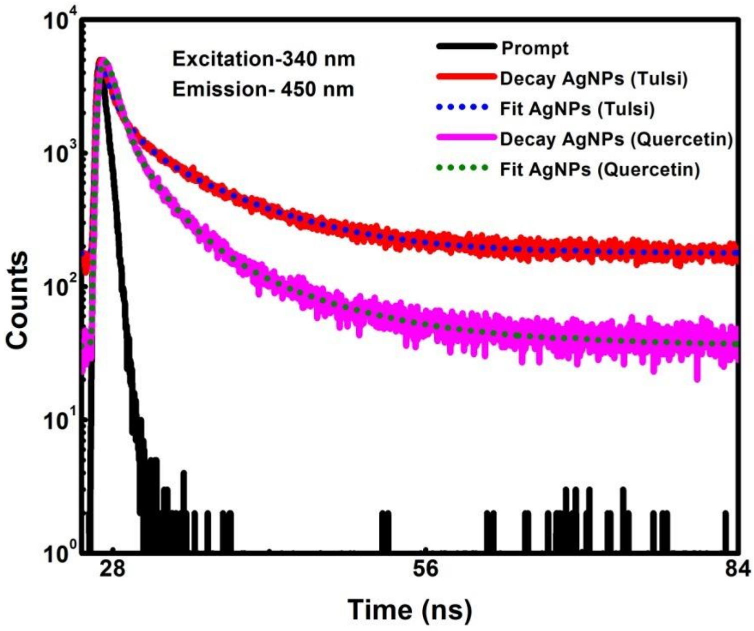

Figure S4: PL decay profiles of AgNPs in water prepared using Tulsi extract and quercetin as precursors.
